# Virtual Nursing Pilot in the Inpatient Setting: Qualitative Evaluation

**DOI:** 10.2196/76994

**Published:** 2026-03-19

**Authors:** Ksenia Gorbenko, Maria Bailon, Jordan Randall, James Viskochil, Clara Cualing, Patrick Healy, Madhu Mazumdar, Robert Freeman

**Affiliations:** 1 Department of Population Health Science and Policy, Icahn School of Medicine at Mount Sinai, One Gustav Levy Place, Box 1077, New York, NY, 10029, United States, 1 212-659-1470; 2 Windreich Department of Artificial Intelligence and Human Health, Icahn School of Medicine at Mount Sinai, New York, NY, United States; 3 Institute for Healthcare Delivery Science, Department of Population Health Science and Policy, Icahn School of Medicine at Mount Sinai, New York, NY, United States; 4 Medical/Surgical Unit, Mount Sinai Hospital, Mount Sinai Health System, New York, NY, United States; 5 Clinical Operations and Analytics, New York–Presbyterian Hospital, New York, NY, United States; 6 Technology, Innovation, and Transformation, Rush University System for Health, Chicago, IL, United States; 7 Quantitative Data Sciences, Mount Sinai Tisch Cancer Center, New York, NY, United States

**Keywords:** virtual nursing, evaluation, qualitative, systems integration, change management, digital transformation, care delivery model, care coordination, staff satisfaction

## Abstract

**Background:**

Global nursing shortages require innovative care delivery models. Virtual nursing is a cutting-edge model being explored.

**Objective:**

This study aimed to examine the perspectives of clinical and administrative staff involved in a virtual nursing pilot on a medical-surgical unit and to identify best practices for future adoption.

**Methods:**

We conducted a qualitative evaluation using individual semistructured interviews with virtual and bedside nurses, nurse executives, and IT project managers implementing a virtual nursing pilot program at a medical-surgical unit with 35 private and semiprivate beds at the Mount Sinai Hospital, a 1110-bed acute care hospital in New York, NY. Interviews took place in the spring of 2024 and were completed via phone or Zoom, audio recorded, and professionally transcribed. Participants were selected using purposive sampling. The authors applied an iterative thematic analysis to transcripts using Dedoose. Claude.ai was used to generate code summaries for select codes.

**Results:**

Of 33 individuals approached, 16 (48.5%) consented to participate. Nine participants were clinical staff (virtual and bedside nurses and nurse managers), and 7 were executive leaders or managers in nursing and informatics. Our analysis identified the following themes: (1) staff attitudes toward virtual nursing shifted from resistance to acceptance over time, (2) direct communication channels between virtual and bedside nurses were critical for efficient care coordination and model adoption, (3) admission and discharge processes evolved throughout the pilot implementation, and (4) adaptable staffing allocations were necessary to accommodate fluctuating patient census and unit demands.

**Conclusions:**

The main beneficiaries of this intervention, bedside nurses, found their virtual counterparts helpful following a few adjustments. Participants reported a perceived reduction in administrative burden, uninterrupted completion of clinical tasks, and they felt their overtime was reduced, which all increased their buy-in for this care model. There are several opportunities for improvement, such as real-time communication, unit-specific virtual nurse training, and flexible staffing for high-volume units. Our findings suggest that virtual nursing can address staffing challenges. Calculating the true return on investment for virtual nursing programs will require comprehensive mixed methods evaluations of such outcomes as care team and patient satisfaction, length of stay, readmission prevention, completion of nursing tasks, and reduction in overtime.

## Introduction

Technological advancements, staffing challenges, and evolving patient expectations are driving a digital transformation that is rewriting how we deliver health care. The COVID-19 pandemic accelerated the adoption of electronic communication platforms to help reduce the spread of the deadly virus. A workforce crisis has emerged globally with the United States facing a shortage of between 1.8 and 3 million nurses according to different projections [1,2]. The excessive workload placed on existing health care personnel has resulted in heightened stress, burnout, psychosomatic disorders, anger, and anxiety [1]. To address this challenge, health care leaders are exploring a variety of digital health tools and integrated care models. The use of telehealth for outpatient visits increased 38 times in the first 2 years of the pandemic [3]. The use of digital tools also increased in the inpatient setting [4]. The majority of these interventions have involved a physician connecting with a patient or bedside staff (a nurse) in a patient’s home or a clinical setting [5,6].

The pandemic’s toll on the health care workforce, nurses in particular, has put new pressure on hospitals to identify nursing tasks that could be completed remotely. Among these are administrative tasks such as admissions and discharges [7], patient education [8], and 1:1 sitting [9]. Of these, virtual sitting for fall prevention has the most evidence, with 12 studies conducted between 2009 and 2021 [9]. Using virtual nurses for admissions and discharges is more recent, with only three virtual nursing pilots on the topic published to date [7,8,10]. Other formats of virtual nursing have also been explored. For example, colleagues from the Mayo Clinic reported on an inpatient virtual registered nurse (RN) program, in which experienced nurses (often retired or nearing retirement) provided guidance to bedside RNs and proactively monitored patients, with no difference in patient safety and care outcomes [11,12].

In a collaborative effort to address staffing challenges and improve patient care, the Mount Sinai Hospital (MSH) partnered with Banyan Medical Solutions, a third-party vendor, to implement a virtual nursing platform on a selected inpatient unit. The implementation process involved installing compatible large-screen smart TVs, wall-mounted cameras, and pillow speakers, enabling virtual nurses to perform critical roles such as patient education, admissions, and discharges. This qualitative research study aimed to examine partner perspectives on this virtual nursing pilot at MSH with a focus on operational implementation and to identify best practices and recommendations for further refinement.

## Methods

### Setting

In January 2024, a virtual nursing pilot was initiated at a 35-bed medical-surgical unit at MSH. The fast-paced unit with high daily discharges accepts various postoperative patients. Virtual nurses, credentialed by the state but working remotely from a command center, have full access to the hospital’s electronic health records. These nurses joined the company in 2021 2022, are relatively junior with experience in other settings, and chose virtual work for personal reasons. Privacy protocols included “virtual knock” protocol, verbal consent requirements, and headphone availability for patients in semiprivate rooms.

### Study Design and Sampling

We conducted a qualitative study using semistructured interviews to examine staff and leaders’ perspectives and experiences related to the virtual nursing pilot program. We used purposive sampling to recruit participants from diverse professional backgrounds, aiming to gain a comprehensive understanding of the program from the frontline to leadership viewpoints.

### Recruitment and Participants

Between February and June 2024, we invited 33 individuals to participate through email outreach, in-person shadowing, distributing flyers in common areas, and collaborating with hospital leadership. Data saturation was reached after completing about two-thirds of the nurse interviews, when new interviews yielded little additional information, following accepted standards in qualitative research [13]. We continued to recruit nonclinical personnel to ensure diverse perspectives.

### Data Collection

Semistructured interviews were conducted via Zoom using a MSH account, lasting about 30 minutes each. Topics included patient and staff safety, privacy, technology reliability, program acceptability and usefulness, and improvement opportunities. The interview guide was developed based on research team discussions, particularly input from nursing leaders attuned to staff hopes and expectations. Our interview guide did not specifically explore formal clinical governance structures, focusing instead on operational implementation experiences. Using an interview guide helped ensure consistency and reliability of collected data across participants [14]. Interviewers (JR, MB, KG) used probes for clarification and conducted clinical shadowing (JR, MB) with field notes.

### Data Analysis and the Use of Artificial Intelligence

Interview data were professionally transcribed and analyzed using iterative thematic analysis [15]. The team used Dedoose [16] for data analysis and management, and Claude.ai [17] to generate drafts of data summaries for selected codes. Three researchers (KG, MB, JR) read and coded the transcripts manually, using both deductive and inductive codes, refining codes through weekly discussion. Two coders applied the final codebook to each transcript in Dedoose. Major codes included care coordination, impact on workload, opportunities for improvement, and inclusion in decision-making. Minor codes included staff satisfaction, timing, best practices, and limitations of virtual nursing. After coding, the team identified key themes, downloaded relevant excerpts, and used AI to generate comparative summaries for each subtheme. Three analysts (KG, JR, MB) combined, iterated, and edited these summaries to create drafts of Results sections for further team refinement.

### Ethical Considerations

The study was determined to be exempt by the Program for Protection of Human Subjects at the Icahn School of Medicine at Mount Sinai on January 17, 2024 (STUDY-23-01378). Informed consent was obtained after the nature and possible consequences of the study were explained. The study adhered to local, national, regional, and international law and regulations regarding protection of personal information, privacy, and human rights. The study was deemed exempt by MSH’s Human Subjects Protection Program, with all participants consenting to be interviewed and recorded, and receiving a US $25 gift card as compensation.

## Results

### Participants

Of the 33 individuals invited, 16 (48.5%) staff members consented and completed interviews (Table 1). The participants included 9 clinical staff (RNs, nurse managers, and virtual nurses) and 7 nonclinical personnel (executives in nursing and informatics, and IT managers). Based on the analysis of the data, we identified the following 4 major themes: (1) staff attitudes toward virtual nursing shifted from resistance to acceptance over time; (2) direct communication channels between virtual and bedside nurses were critical for efficient care coordination and model adoption; (3) admission and discharge processes evolved throughout the pilot implementation; and (4) adaptable staffing allocations were necessary to accommodate fluctuating patient census and unit demands.

**Table 1.** Participant characteristics and professional background (N=16).

Participant	Count, n
Professional background	
Registered nurse, including virtual nurses and executives with nursing background	12
Information technology or managers	3
Executives, including MSHS^a^ executives and Banyan	2
Professional role	
Registered nurses at bedside	7
Virtual nurses	3
Information Technology	2
Operations personnel	2
Executives, including MSHS executives and Banyan	2
Gender	
Female	14
Male	2

a MSHS: Mount Sinai Health System.

### Theme 1: Implementation Process: Staff Perspectives Adapt From Resistance to Acceptance

The implementation of virtual nursing revealed a clear evolution in staff attitudes, moving from initial skepticism to overall acceptance. Some of it had to do with the timing and depth of staff involvement in virtual nursing implementation decisions. Staff accounts indicate early concerns about role displacement and workflow disruption (Table 2, example 1a), which were exacerbated by the fact that early-stage participation was limited and staff were brought in late in the planning process. Staff identified missed opportunities for leadership to proactively engage frontline workers: “I would have loved to have had that interaction with Banyan earlier, because we had a bunch of questions that our people didn’t have answers to” (Bedside RN, CL01; Table 2, example 1b). This delayed engagement led to accumulated questions and uncertainty among staff, though many issues were eventually resolved through concentrated information sessions. Nurse participants also reported that once the training was complete, they were relieved and realized that virtual nurses could be helpful (Table 2, example 1c).

**Table 2.** Key themes and representative quotes from participant interviews. Theme 1: Implementation process.

Example No.	Subtheme	Quote and participant role, ID
1a	Initial fears	“So there was a lot of trepidation. what is this actually going to look like. like physically how are they going to do this? And they’d be like, oh, we’ll get back to you.” (Bedside RN, CL01)
1b	Staff involvement	“.if the leadership is like. we’d like to go talk to the staff before we officially decide this so that we can let them know exactly how this works, maybe they’ll be a little bit more comfortable with it” (Bedside RN, CL02, Table 2, 1b).
1c	Relief	"We were thinking it was going to be a thing that we would have to worry about. But once you know all you have to do is a few clicks in the Banyan board, then I think that is definitely really helpful.” (Bedside RN, CL03)
1d	Decisions made at leadership level	“Because we are a union hospital, we did have to be very specific. That came from the CNO and the union.” (Nursing executive, NCL06)
1e	Leadership and change management	"It requires multiple conversations and sessions in advance of the model being implemented for people to first understand why we’re doing this. It's not for the heck of it. It's to help you" (Nursing executive, NCL07)
1f	Peer-to-peer learning	"I think if also there was an opportunity for people from [our unit] to go talk to the other units about it and how it worked, like to hear from peer-to-peer, that would be really helpful" (Bedside RN, CL03).

Several bedside nurses noted they wanted to be involved in technology evaluation and workflow decisions. This, however, was not always possible, as we learned from the interviews with nursing executives and nurse managers. One consideration was that the union leaders’ agreement on what tasks would be delegated to virtual nurses. Union considerations shaped decision-making authority, with decisions made at the leadership level (Table 2, examples 1c and 1d).

Our analysis also demonstrated real-world challenges in achieving meaningful staff participation while maintaining unit operations. Leaders described efforts to include staff while acknowledging limitations: "We do pull nursing staff off the floor to assist with any projects to get their insight, but we often know that they’re not able to stay every time for the whole hour, if at all” (Executive leader, NCL06). This resulted in a selective approach to staff involvement, with few staff members being able to go onsite to another hospital to observe Banyan operations.

During interviews, leaders emphasized their commitment to address staff concerns and support adoption of the new system. As one operations leader explained, "I would do anything I could to get leadership to the unit, to recognize that we appreciate you... we’re doing this as a resource for you” (Executive leader, NCL04). The implementation process required careful messaging and consistent support (Table 2, example 1e).

Finally, peer influence played a crucial role in shifting perceptions, particularly as floating nurses shared positive experiences across units: "When we get nurses from other units that float to us, they’re like oh my god, this is amazing. They all love it” (Bedside RN, CL06). Initial resistance transformed through hands-on experience and peer testimonials, with staff gradually recognizing virtual nursing as supportive rather than replacement technology (Table 2, example 1f).

### Theme 2: Optimizing Technology Integration: Opportunities and Best Practices

System reliability and integration emerged as critical factors in implementing a virtual nursing platform. Here we describe *several operational opportunities for improvement* that can be used to establish best practices for implementation. These included technical integration to eliminate delays in communication, resolving audio quality issues, remote printing capability for virtual nurses, and volume control in shared rooms. These technical challenges collectively highlight the importance of robust infrastructure and seamless integration between systems for optimal integration into operational workflows.

*Integration with existing hospital systems* presented ongoing challenges. Multiple nurses noted issues with electronic health record connectivity and documentation access. These integration issues required bedside staff to navigate between multiple platforms, creating workflow inefficiencies (Table 3, example 2a). Bedside nurses desired a more nuanced communication tool with their virtual nurse partners in which they could specify discharge timing, for example, when transportation for the patient was already prearranged, and a direct communication line between virtual nurses and providers. This highlighted the need for systems that could accommodate real-time coordination between virtual and bedside teams.

**Table 3.** Key themes and representative quotes from participant interviews. Theme 2: Optimizing technology integration.

Example No.	Subtheme	Quote and participant role, ID
2a	Systems integration	"Opening a separate screen, it sounds crazy, but it's often a pain... We basically have to open up a separate page. We have to go out of our Epic and open up a separate page to move Banyan." (Bedside RN, CL04)
2b	Audio quality	"It's loud... depending on the age group, depending on how sedated the patient is... Some of it is a [local] issue, that some of the TVs aren't coming through the call bell." (Bedside RN, CL04)
2c	Printing connectivity	"...if [virtual nurses] have completed all the discharge… it's just… one extra button to print it. Whereas if we have to print it, we have to go in, we have to look at all the things, then print it, and then gather it again. So it just saves a couple extra minutes with it being like oh, you're already in that section, you press the button.” (Bedside RN, CL02)
2d	Patients with special accommodations	“So at that point I would probably do the interpretive services myself in person because on top of it that there's a new barrier of virtual, we have to then find an interpreter, and then we have the technology issues of whether the phone in the room is actually working... But even when it is working, I've seen that admission, instead of taking probably like a half an hour, it takes like over an hour.” (Bedside RN, CL05)
2e	Workarounds	“And they themselves, taxi services, most of the time they’re rude… they’re like no, if they’re not here in the next two minutes I have to leave and pick up the next person. They don’t care. And then we’re like let’s just do the discharge ourselves and let’s just… like let’s not even wait for transport, let’s just roll the patient down in the wheelchair on our own before they lose their taxi.” (Bedside RN, CL05)

The interoperability challenges between the virtual nursing platform and the hospital’s EHR system affected *care plan documentation and continuity*. Virtual nurses documented within both the Banyan system and Epic, but the lack of seamless integration meant that care plan updates were not always immediately visible to bedside staff without actively switching between systems. When virtual nurses completed admission assessments or discharge education, bedside nurses reported needing to actively seek out these updates rather than receiving automatic notifications of completed tasks. Remote printing connectivity for virtual nurses represented another technical hurdle. Bedside nurses explained that efficiency could be gained through improved printing functionality (Table 3, example 2c). According to one nurse, “it just saves a couple extra minutes… [if] you’re already in that section, you press the button” (Bedside RN, CL02). While virtual nurses tried to send paperwork to print locally on the unit, this functionality did not work at the time of the interviews, making bedside nurses sign into the electronic medical record each time for the (virtually) discharged patient, find all the information, and send it to the printer again.

*Audio quality issues* particularly affected patients’ willingness to accept a virtual nurse and staff efficiency, with problems being more pronounced during night shifts. Volume control concerns in shared rooms further complicated the situation, creating potential privacy issues and patient dissatisfaction, highlighting the need for better audio privacy solutions such as mandatory headphone use or room-specific volume controls (Table 3, example 2b).

While adaptations in the virtual nursing program showed promise for standard patient interactions, additional complexities arose with specific patient populations. Extended processing times for patients requiring interpreter services emerged as a particular challenge. Our bedside nurse participants reported foregoing virtual support altogether to save time and ensure effective communication with patients who needed interpreter services (Table 3, example 2d).

In response to these challenges, staff developed various *workarounds* to maintain efficient patient care. For example, bedside nurses described how transportation constraints sometimes necessitated bypassing virtual nursing protocols. One nurse explained that taxi drivers often gave ultimatums about immediate departure, stating they would leave if patients weren’t ready. This led staff to expedite discharges independently rather than risk patients losing their transportation (Table 3, example 2e). This adaptive approach allowed staff to balance the benefits of virtual nursing with practical operational needs. Another workaround—the implementation of Epic’s secure chat functionality—marked a significant improvement in real-time coordination, with staff reporting it helped them coordinate plans with their virtual counterparts. Developing clear communication protocols, particularly for urgent situations, proved essential for maintaining seamless patient care across virtual and bedside teams.

IT specialists in our study highlighted the importance of technical considerations, such as future *interoperability*, beyond the immediate virtual nursing pilot. One IT leader pointed out that it is important to consider the use/reuse of technology components, such as smart TVs, for new nursing workflows that could be covered remotely: “how do we utilize a single or a smaller set of devices?” (NCL03). This perspective underscores the importance of strategic planning for technology integration that considers both current needs and future applications.

### Theme 3: Care Coordination: Benefits of Virtual Nursing on Teamwork

The implementation of virtual nursing transformed admission and discharge processes, creating new team dynamics between bedside and virtual nurses. This theme explores how these workflows evolved and the benefits that emerged from this collaborative approach.

#### Transformation of the Discharge Process

Before the virtual nursing pilot, discharges required bedside nurses to manage multiple competing priorities simultaneously. One nurse described the traditional discharge process prior to the virtual nursing pilot implementation that involved educating patients, who were often frightened, about their discharge, trying to be supportive, “all while maybe a call bell’s ringing, you’re trying to get somebody their meds, you’re hopefully not getting an admission because your workload is full” (Bedside RN, CL04; Table 4, example 3a). With the introduction of virtual nursing, the process became more streamlined. The same nurse explained that now they just switch a certain setting and “Banyan will often just start doing the discharges” (Bedside RN, CL04), freeing bedside staff to perform other tasks.

**Table 4.** Key themes and representative quotes from participant interviews. Theme 3: Care coordination.

Example No.	Subtheme	Quote and participant role, ID
3a	Old workflows (previrtual nursing)	“…prior to Banyan, you waited for a discharge order… You printed out your paper and we went to the bedside… we’re a postsurgical unit, many people are absolutely frightened about going home with a new hip… and you would spend a tremendous amount of time educating, and teaching, and explaining their discharge. Then we wait for transport. And this is all while maybe a call bell’s ringing, you’re trying to get somebody their meds, you’re hopefully not getting an admission because your workload is full.” (Bedside RN, CL04)
3b	Good catch	“The only time I remember that it didn’t go well is we had a patient who needed… [Lovenox] education... And the only way I was able to figure that out is because he was leaving and then his wife came up to me and she was like we never got the Lovenox education… So I think the nurse should have picked it up, like oh, you’re going home on Lovenox injection, do you know how to administer it?.. He could have just walked out, went home, and not know how to do it. Good thing his wife [asked]… And I was like oh, okay, let’s go back into the room and we will go through the steps on how to administer the Lovenox injection.” (Bedside RN, CL06)
3c	Division of patient education responsibilities	"Especially with our ortho patients, they are usually prescribed a certain blood thinner so that they don't develop any clots, and if they're already taking blood thinners at home, and there's an issue with the medication or any interaction, they could definitely help somebody, you know, prevent them from having an adverse reaction..." (Bedside RN, CL05) "The hands-on teaching, plus the discharge, can take a long time. So for the discharge paperwork to be reviewed, and then I come in and just have to do Foley teaching, for example, with the leg bag, that just takes a lot of time off of my plate for me to focus on other things." (Bedside RN, CL03)
3d	No direct communication with providers	“[Virtual nurses] are not able to communicate with the frontline providers, and if… the patient has any questions, and they can’t answer, and then they’ll message us, and then I have to message the doctor, and then I have to relay that information to the virtual nurse… So, you know what I mean, so she still has to go through me…” (Bedside RN, CL06)

The discharge process transformation remains a work in progress, particularly regarding the division of educational responsibilities between virtual and bedside nurses. This was evident when a patient nearly left without critical Lovenox self-injection education, discovered only when his wife proactively inquired as they were leaving (Table 4, example 3B). Following this learning opportunity, the nurse implemented a new protocol of personally reviewing the After Visit Summary before handoff, proactively identifying teaching responsibilities that fall within the bedside nurse’s scope rather than the virtual nurse’s capabilities, thus ensuring patients receive all necessary education before discharge.

#### Emerging Communication Patterns and Task Coordination

As the unit grew more comfortable with virtual nursing, new communication patterns emerged between bedside and virtual nurses. Bedside nurses noticed that virtual nurses began to proactively update them on task completion. Virtual nurses reported developing strategies for prioritization through communication with bedside staff. As one virtual nurse explained: "Communication is key. You just let the bedside nurse know what’s going on… could you please… let us know the order that you want us to prioritize the patients, whose transportation is coming first… It makes all the difference in care” (Virtual RN, CL08). Virtual nurses reported initial lack of clarity about their role among patients, with one participant noticing an improvement in that respect: “the patients [are] just being educated more… regarding our services” (Virtual RN, CL07).

The division of educational responsibilities between virtual and bedside teams emerged as a key strength of the program. The system proved particularly valuable for complex cases, including newly diagnosed patients requiring extended education sessions. One bedside nurse highlighted the importance of virtual nurses’ role in patient education, particularly for high-risk patients. She explained how orthopedic patients on blood thinners need to have their medications at home reviewed in case they were already taking blood thinners to prevent any interactions or adverse reactions (Table 4, example 3c).

A gap that remained unresolved at the time of the interviews had to do with virtual nurses’ inability to message providers. For example, if a patient had a question during discharge that the virtual nurse could not answer, they had to message a bedside nurse who would then message a provider and report back to the virtual nurse who would relay it to the patient (Table 4, example 3d). Being unable to communicate directly with providers made this process unnecessarily involved and inefficient.

#### Time Efficiency and Focus on Direct Patient Care

A significant benefit reported by bedside nurses was how virtual nursing saved them time and allowed them to focus on hands-on teaching and direct patient care. One bedside nurse mentioned that discharges traditionally could take up to an hour and were now taken care of by their virtual counterparts (Table 4, example 3c). Another nurse echoed: “We’re not stuck in a room for an hour doing admission questions and then having four other patients call on the Vocera and then get interrupted, going back and forth. That’s what was happening before” (Bedside RN, CL06).

The ability to handle multiple tasks simultaneously was particularly valuable during busy periods. As one nurse explained: “Because if you’re getting two admissions and you have a discharge, it’s just a lot… So it’s really helpful because then you’re able to actually focus on hands-on patient care as opposed to…the paperwork aspect of it” (Bedside RN, CL03).

#### Improved Nursing Satisfaction and Work-Life Balance

The collaboration between virtual and bedside nursing staff resulted in positive outcomes for staff satisfaction and work-life balance. One nurse remarked, "It’s nice to just like have that thought taken off your plate, as well as the admissions, just not having to stay late to do an admission when they come at… poor timing.” (Bedside RN, CL02). Overall, the evolution of teamwork between virtual and bedside nurses created a more efficient workflow for admissions and discharges, allowing nurses to dedicate more time to direct patient care while ensuring comprehensive education and documentation.

### Theme 4: Timing Is Key: Staffing Capacity and Patient Flow

Workflow integration and staffing alignment emerged as critical factors in the uptake and efficiency of virtual nursing, revealing the importance of proper coordination between virtual and bedside teams in managing patient flow.

#### Managing High-Volume Patient Movement

The partnership between virtual and bedside teams impacted patient workflow management, particularly during high-volume periods. One bedside nurse explained that their unit had “a colossal amount of movement… [with] 14 to 20 [daily] discharges and admissions” (CL04). Effective workforce distribution proved essential for managing such volume, as staff needed to adapt to fluctuating demands throughout the day. As another bedside nurse explained: “Sometimes they could say 10 [discharges], so they have, let’s say, 5 [virtual] nurses on board, but then as the day goes on, the discharges do happen, so instead of having 10 we’ll have 18” (Bedside nurse, CL06). This unpredictability highlighted the ongoing need to develop flexible staffing management strategies that could respond to rapidly changing conditions. Ascertaining the right staffing levels for virtual took some tweaking, as is evidenced by one bedside nurse report about initial challenges during the overnight shift, when there was only one virtual nurse available (Table 5, example 4A).

**Table 5.** Key themes and representative quotes from participant interviews. Theme 4: Timing is key.

Example No.	Subtheme	Quote and participant role, ID
4a	Initial insufficient staffing	“…it also comes down to how many people, how many nurses is Banyan hiring for our unit per shift... Because the thing is, [my supervisor] said oh, we noticed that things are running slow. [The virtual nurse said] I’m sorry, I apologize, it’s just [I am] the only nurse with them at that shift… That was when Banyan was first implemented… I actually think Banyan can work in a fast-paced, but if there’s enough staff, virtual nurse staff, and also to make things run faster.” (Bedside RN, CL05)
4b	Impact on hospital throughput	"Once we discharge this patient we can pull more patients coming from the PACU and from the ED, so it helps them, like don't hold the OR schedule nor have a critical diversion in the ED" (Bedside RN, NCL05).
4c	Nurses more readily accept admissions at end of shift	“I think now that we have the Banyan nurses, nurses are not... reluctant on taking the admission from PACU. They'll just pick up because they know that when they come onto the floor that they don't have to deal with these admission questions, that they're going to be taken care of” (Bedside RN, CL06).

#### Impact on Hospital-Wide Operations

The virtual nursing system’s impact extended beyond individual patient care to affect broader hospital operations. The most positive impact of the pilot, as perceived by our participants, was what they described as improved patient throughput. During peak periods, virtual nurses supported bedside teams, creating capacity to process patient admissions and discharges simultaneously. High volume peaks required different coordination compared to slower-paced environments, while night shift operations needed special adaptations to maintain patient comfort and prevent disruption during quiet hours. According to one nurse, by facilitating more efficient discharges, the system improved patient throughput across multiple departments (Table 5, example 4b).

This improvement in patient flow was also reflected in staff attitudes toward accepting new admissions. Nurses used to be reluctant to accept an admission from PACU, for example, if it meant they would have to stay longer after the end of their shift to complete it. According to several participants, this was no longer the case (Table 5, example 4c).

#### Time Investment in Patient Education

Virtual nurses reported dedicating significant time to patient education, highlighting the value of this investment despite its potential impact on short-term throughput. One virtual nurse reflected on the depth of engagement required: "I would say I think I’m long with discharges personally… I take a while. They have so many questions… The family’s in there, the mom, the dad. So yeah, I feel like my discharges are long, like an hour… I feel bad. Some of them are newly diagnosed, so that’s even longer, like an hour and a half” (Virtual RN, CL09). This time investment, while potentially extending the discharge process itself, ultimately supported more comprehensive patient education and potentially better postdischarge outcomes, demonstrating that flexibility in staffing and timing considerations needs to balance efficiency with quality of care.

## Discussion

### Principal Findings

This is the first qualitative evaluation of a virtual nursing pilot on an inpatient unit. Our findings demonstrate that virtual nursing implementation requires careful attention to technological infrastructure, operational readiness, and change management. We identified four major themes that characterized the virtual nursing implementation in our pilot: (1) achieving staff buy-in takes time and can benefit from earlier engagement strategies; (2) seamless technology integration, especially direct communication channels, is critical for smooth operation; (3) recognition of virtual nurses as integral team members, rather than auxiliary support, enhances collaborative care delivery; and (4) virtual nursing coverage must meet the demand to maintain relevance within the organizational or unit context.

Our study offers insights into implementing virtual nursing on inpatient units, which other hospitals can use when considering similar models. The unique unit or patient population characteristics—high volume, fast pace, surgical patients with diverse diagnoses—fit well with the virtual nursing pilot seeing benefits in admission or discharge support and patient education. If virtual support was unavailable or inefficient, the bedside nurses reverted to their traditional workflows. With the exception of some initial issues, the vendor was able to meet the challenge and provide sufficient support. Virtual nursing implementation on slower-paced units might prioritize functions like virtual sitters, which could be particularly useful on observation units.

Our findings highlight the critical importance of seamless EHR integration for virtual nursing models. The fragmented documentation workflow we observed, where staff navigated between multiple systems, created inefficiencies that could impact care continuity. The care plan coordination issues, exemplified by the Lovenox education incident, demonstrate how documentation gaps can directly affect patient safety. Virtual nursing programs must establish clear protocols for documenting care plan handoffs, particularly for complex patients requiring both virtual and bedside interventions and prioritize robust interoperability that allows direct documentation within the primary care record.

Technical and system reliability directly impacted operation readiness. The integration between EHR and the virtual nursing platform, audio quality, and physical environment considerations influenced staff adoption and clinical workflows. Virtual nursing adoption hinges on infrastructure investment that could be a barrier for smaller hospitals.

The pilot used out-of-state junior RNs. Other staffing models could be considered, such as floating bedside nurses as virtual for a fraction of their time. Implementers should weigh the efforts needed to educate new virtual staff vis-à-vis managing virtual nursing in-house.

Flexible staffing in response to patient flow emerged as one of the most critical factors for optimal operation. Implementation followed an overall positive trend—initial increases in workload during rollout, followed by productivity improvements once frontline staff adapted to their new workflow. Participants reported positive downstream effects such as reducing operating room holds and emergency department diversions through improved time to discharge and bed turnover.

This pilot aligns well with the cutting-edge innovations in health care operations. Our findings demonstrate that implementation depends on structured approaches to change management [18] while minimizing disruption to care delivery. Key insights from our analysis—albeit not new [19]—reveal the importance of early staff involvement in the decision-making process.

Staff adoption of the virtual nursing platform followed a progression from initial skepticism to approval. Clinical champions were powerful influencers of technology acceptance. Clear role separation created effective partnerships, particularly in educational delivery. Virtual nursing could leverage experienced nurses’ expertise post COVID-19 pandemic, like programs at other institutions have shown [11,12].

### Limitations and Strengths

Our study has the following limitations. We interviewed hospital personnel but not patients or caregivers. Understanding how both patients and caregivers view this intervention is essential for improving it further. Researchers need to examine not just how the interactions between patients and virtual nurses are designed to work, but also how patients and caregivers actually experience and respond to these interactions. It is a single-center pilot study in an academic medical center; our findings might not apply to a different health care setting or patient population. With about a 50% response rate, a nonrespondent bias is possible. Our study did not include quantitative data on reduced overtime, improved throughput, or quality indicators such as patient satisfaction, medication errors, or readmission rates. All comments related to these important metrics were based on the impressions of bedside and virtual staff and executive leaders. We also did not systematically examine formal clinical governance models or committee structures that may have guided the implementation process, nor did we evaluate preimplementation preparation or formal support models during rollout. However, this is the first published qualitative study of virtual nursing programs to our knowledge, capturing valuable nurse and leadership perspectives through its qualitative approach.

### Recommendations for Planning Virtual Nursing Implementation

Based on our findings, implementation of virtual nursing requires attention across several domains. Organizations considering adoption should prioritize early infrastructure investment, unit-specific workflow design, clear governance structures, and intentional patient-facing communication. The following checklist sums up key considerations to guide planning and implementation.

- Technical infrastructure
-  Invest in technical and communication infrastructure upfront to ensure seamless system integration between virtual and bedside nurses and between virtual nurses and providers
-  Develop or adopt AI tools to support automatic patient prioritization for admission and discharge

- Workflow
-  Implement dynamic staffing allocations responsive to patient volume fluctuations
-  Adapt and optimize workflows to reflect specific demands of each unit
-  Develop clear standard operating procedures for communication between virtual and bedside staff
-  Conduct regular review of staffing allocations based on utilization patterns

- Clinical governance
- Establish formal governance structures with designated clinical champions

- Patient-facing communication
-  Use nurses’ insights to create standard messaging on introducing the virtual nursing program to patients and setting expectations
-  Develop privacy standard operating procedures for shared rooms, including headphone distribution and consent procedures for virtual interactions

### Conclusions

Virtual nursing is a cutting-edge care delivery innovation that goes beyond adoption of new technology. This virtual team model enhances care coordination, patient throughput, and staffing while keeping care patient-focused. Program success requires resources, technical infrastructure, and clear, unit-specific communication. Health care should see it as bedside care extension, with careful planning and ongoing adjustments based on data and feedback.

This study provides valuable insights for hospital leadership and management to understand the impact on clinical workload, staff acceptability and satisfaction, and patient care quality to support informed implementation decisions. Future research should explore several questions that emerged during this implementation: (1) How do virtual nursing models impact nurse retention and recruitment in a high turnover environment? (2) How might AI integration enhance the efficiency of virtual nursing documentation and communication? (3) How do patients perceive virtual nursing and how can their experience be improved? (4) What does virtual nursing affect quality and safety outcomes such as patient satisfaction, readmissions, and medication errors? Addressing these questions will further develop the evidence for this emerging care delivery model.

## Abbreviations

MSH: Mount Sinai Hospital
RN: registered nurse
